# *PLA2G4F* is a metabolic checkpoint in triple-negative breast cancer: Insights from multiple omics analysis and experiments

**DOI:** 10.1016/j.omton.2025.200963

**Published:** 2025-03-07

**Authors:** Xun Tang, Ning Jiang, Yingying Kou, Shan Cheng, Feng Yan

**Affiliations:** 1 Department of Clinical Laboratory, the Affiliated Cancer Hospital of Nanjing Medical University and Jiangsu Cancer Hospital and Jiangsu Institute of Cancer Research, Nanjing 210009, China; 2 Jiangsu Key Laboratory of Molecular and Translational Cancer Research, the Affiliated Cancer Hospital of Nanjing Medical University and Jiangsu Cancer Hospital and Jiangsu Institute of Cancer Research, Nanjing 210009, China; 3 Good Clinical Practice Office, the Affiliated Cancer Hospital of Nanjing Medical University and Jiangsu Cancer Hospital and Jiangsu Institute of Cancer Research, Nanjing 210009, China; 4 Department of Infectious Disease, Children’s Hospital of Nanjing Medical University, Nanjing 210008, China

**Keywords:** MT: Regular Issue, triple-negative breast cancer, metabolic reprogram, single-cell RNA sequencing, *trans*-omics analysis, PLA2G4F

## Abstract

Metabolic reprogramming is an emerging hallmark and promising therapeutic target in cancer, fueling malignant cells and regulating the tumor microenvironment as a metabolic checkpoint. Triple-negative breast cancer (TNBC), an aggressive subtype with poor prognosis, is marked by high recurrence rates and chemotherapy resistance. However, the metabolic heterogeneity and cell-specific metabolic profiles in TNBC remain underexplored. In this study, we analyzed the expression and prognostic impact of 87 metabolic pathways involving 1,668 genes, identifying 104 candidate metabolic checkpoint genes. Using consensus clustering, we uncovered two distinct metabolic subclusters of TNBC patients that exhibited significant differences in survival. We further characterized the differentially expressed genes, mutation profiles, and microenvironmental features between these two clusters. Additionally, *trans*-omics analysis of single-cell RNA sequencing revealed that *PLA2G4F*, a gene specifically expressed in malignant cells, functioned as a cell-autonomous metabolic reprogramming factor. We validated that *PLA2G4F* promotes the proliferation, migration, and survival of TNBC cells *in vitro*, driven by dysregulated glucose and lipid metabolism. These processes were mediated, at least in part, through the activation of the *AKT/PI3K* signaling pathway. This study highlights the metabolic heterogeneity in TNBC and identifies *PLA2G4F* as a pro-tumor factor, suggesting it as a potential novel therapeutic target.

## Graphical abstract

**Figure:**
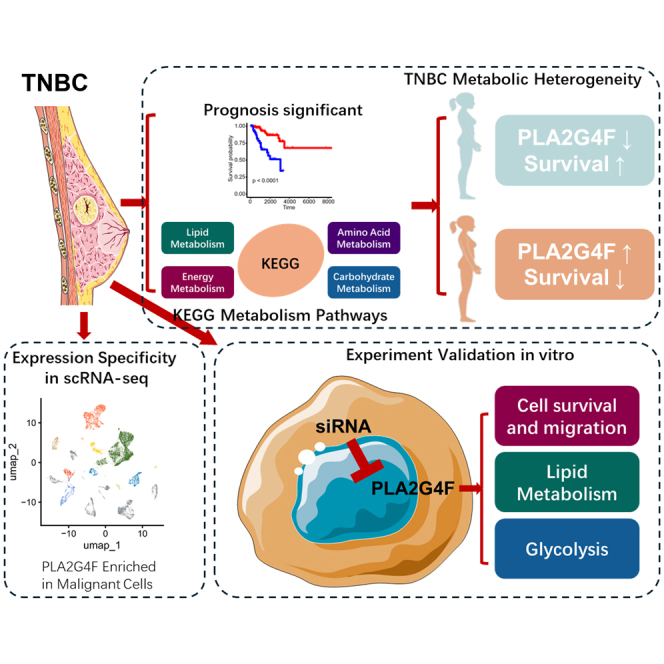

## Teaser

This study uncovers metabolic heterogeneity in triple-negative breast cancer (TNBC) and identifies PLA2G4F as a metabolic checkpoint gene driving tumor progression through glucose and lipid dysregulation. By integrating multi-omics and single-cell analyses, it highlights PLA2G4F’s role in metabolic reprogramming, providing insights into TNBC pathogenesis and revealing a novel therapeutic target.

## Introduction

Metabolic reprogramming, an emerging hallmark of cancer, plays a critical role in various aspects of tumor progression, including triple-negative breast cancer (TNBC).^1^^,^^2^ Growing evidence has demonstrated that tumor metabolic reprogramming occurred in both malignant and non-malignant cells within the tumor microenvironment, forming a comprehensive metabolic regulatory network with distinct cellular metabolic signatures.^3^^,^^4^^,^^5^ The altered metabolite profiles not only modulated energy dynamics but also orchestrated signal transduction in tumor microenvironments, creating a vicious cycle in tumor progression.^5^^,^^6^^,^^7^ Beyond the local tumor site, the intricate reprogramming of tumor metabolism can exert far-reaching consequences on the host’s systemic metabolic landscape, potentially leading to cachexia and impaired antitumor immunity.^8^^,^^9^^,^^10^^,^^11^ Targeting metabolism is increasingly regarded as a promising approach in cancer treatment, further defined as a metabolic checkpoint. GPR31, serving as a sensor for citric acid cycle intermediates, has been reported to promote cancer development and regulate immune responses.^7^

TNBC, defined by the absence of estrogen receptor (*ER*), progesterone receptor (*PR*), and human epidermal growth factor receptor 2 (*HER2*) expression, exhibits a distinct clinical course characterized by high aggressiveness, early recurrence, and poor prognosis, presenting a significant challenge in clinical oncology.^12^ Although TNBC patients benefit from surgical intervention, they still experience high recurrence rates and chemotherapy resistance in subsequent treatments.^13^ Thus, there is an urgent need to understand the molecular pathological signatures of heterogeneous TNBC, which can be developed into novel therapeutic targets. One promising avenue is the exploration of metabolic gene signatures. Indeed, TNBC has been previously reported to exhibit metabolic heterogeneity and targeting several metabolic genes could inhibit the tumor progression as well as regulation tumor microenvironment.^14^ Reduced expression of ACC2, a critical fatty acid synthesis (FAS) gene, is indicative of increased fatty acid oxidation (FAO) that leads to breast tumor aggressiveness and is linked with worse prognosis and outcomes in TNBC cohorts.^15^ One of the limitations was that the current researchers usually focused on the metabolism at bulk or single-cell level independently, which might lead to the gap of comprehensive understanding of cancer metabolism. Moreover, since malignant cells are primarily responsible for reprogramming tumor microenvironment metabolism, candidate targets should ideally be specifically expressed in these cells. Therefore, characterization comprehensive cell-specific metabolic reprogramming in TNBC is urgently required.

In this study, we systematically analyzed the prognostic impact of the metabolic gene landscape in TNBC. Through consensus clustering, we identified two distinct metabolic subclusters of TNBC patients that were significantly associated with prognosis, lipid metabolism, and the tumor microenvironment. *Trans*-omics analysis of independent single-cell RNA sequencing (scRNA-seq) data identified *PLA2G4F* as a promising target, specifically expressed in malignant cells. The oncogenic effects of *PLA2G4F* and its role in reprogramming lipid and glucose metabolism were validated *in vitro*. Our study offers a comprehensive understanding of metabolic abnormalities in TNBC at both the bulk and single-cell levels, emphasizing the potential of metabolic reprogramming as a therapeutic strategy.

## Results

### Metabolic heterogeneity in TNBC patients

**Figure 1:**
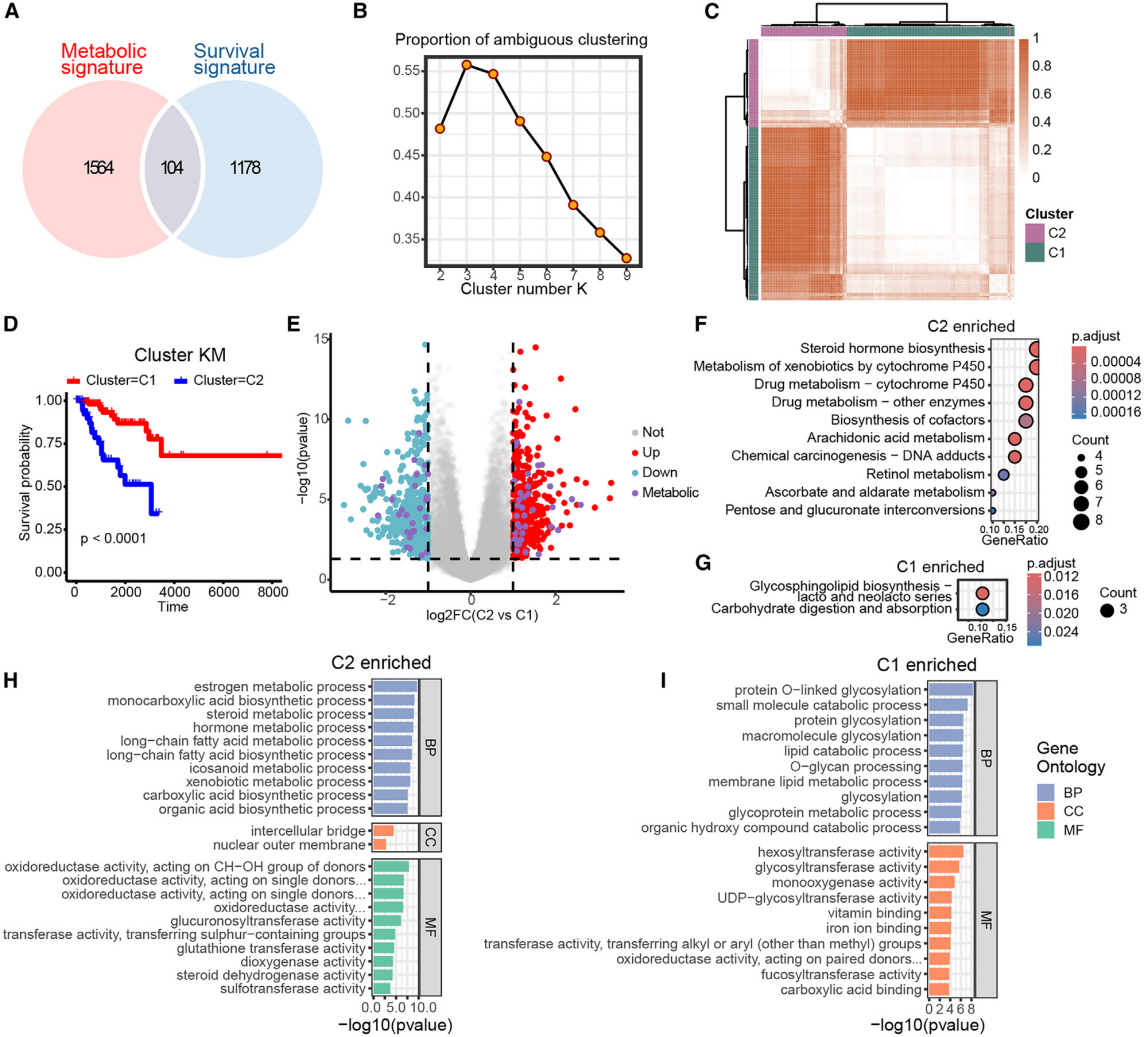
Metabolic heterogeneity was crucial for TNBC prognosis (A) Venn diagram showing the overlap between metabolic genes and survival-related genes in TNBC. (B) The proportion of ambiguous clustering (PAC) score, where a lower PAC value indicates a flatter middle segment, facilitating the determination of the optimal k (k = 2) by identifying the lowest PAC and minimizing information redundancy. (C) Consensus score matrix for all samples when k = 2, with higher consensus scores between two samples suggesting a greater likelihood of being clustered together across different iterations. (D) Kaplan-Meier (KM) plot for TNBC patients stratified by metabolic subclusters. (E) Volcano plot illustrating differentially expressed genes (DEGs) in the comparison of C2 vs. C1, with colors representing up-regulated (red), down-regulated (blue), non-significant (gray), and metabolic (purple) genes in TNBC. (F and G) KEGG enrichment dot plots for genes enriched in C2 (F) and C1 (G). (H and I) KEGG enrichment dot plots for genes enriched in C2 (H) and C1 (I).

We began by performing consensus clustering to identify metabolic subclusters among TNBC patients in The Cancer Genome Atlas (TCGA) cohort. Using metabolic genes identified as prognostic through Cox analysis, we selected 104 genes as features for clustering (Figure 1A). The 180 TNBC patients were initially divided into clusters ranging from k = 2 to 9, based on these 104 genes. The cumulative distribution function (CDF) curves and the proportion of ambiguous clustering (PAC) statistics indicated that the optimal number of clusters was k = 2 (Figure 1B). This result was further confirmed by the sample distance matrix (Figure 1C). The two consensus clusters (C1 and C2) exhibited significant differences in survival, with C1 associated with a more favorable prognosis compared with C2 (Figure 1D, *p* < 0.0001).

To further delineate the molecular differences between these clusters, we performed differential expression gene analysis. We identified 411 genes enriched in C2 and 495 genes enriched in C1 (Figure 1E). Kyoto Encyclopedia of Genes and Genomes (KEGG) enrichment analysis revealed that C2-enriched genes were primarily involved in steroid hormone biosynthesis, drug metabolism, and cytochrome P450 pathways, while C1-enriched genes were associated with glycosphingolipid biosynthesis and carbohydrate digestion and absorption (Figures 1F and 1G). Gene Ontology (GO) enrichment analysis supported these findings, with C2 genes linked to estrogen, steroid, and hormone metabolic processes (Figure 1H), whereas C1 genes were enriched in protein O-linked glycosylation, glycosylation, and glycosyltransferase activity (Figure 1I). Notably, both C1 and C2 clusters exhibited reprogramming of lipid and fatty acid metabolic processes, underscoring the critical role of lipid metabolism in TNBC heterogeneity.

### Mutation profile of metabolic subclusters

**Figure 2:**
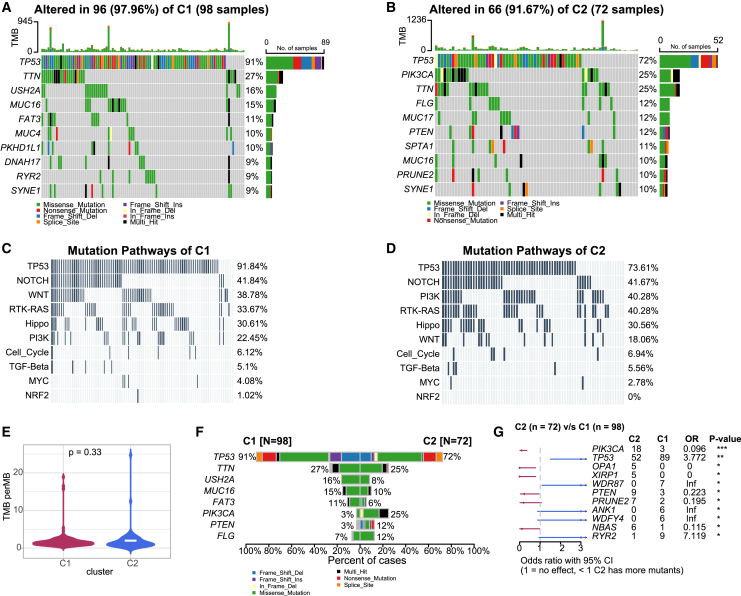
Genetic Characteristics in TNBC Metabolic Subclusters (A and B) Waterfall plots displaying somatic mutations in TNBC metabolic subclusters C1 (A) and C2 (B). (C and D) Waterfall plots illustrating oncogenic signaling pathways in TNBC metabolic subclusters C1 (C) and C2 (D). (E) Violin plot comparing tumor mutation burden (TMB) between TNBC metabolic subclusters. (F) Co-bar plot showing differentially mutated genes between metabolic subclusters. (G) Forest plot of differentially mutated genes between metabolic subclusters.

We next compared the mutation profiles of the C1 and C2 metabolic subclusters. In C1, the top five most frequently mutated genes were *TP53, TTN, USH2A, MUC16,* and *FAT3*, with mutation frequencies ranging from 11% to 91% (Figure 2A). In contrast, the top five mutated genes in C2 were *TP53*, *PIK3CA, TTN, FLG*, and *MUC17*, with frequencies ranging from 12% to 72% (Figure 2B). Analysis of oncogenic signaling pathways revealed that both clusters were enriched in *TP53*, *NOTCH*, *RTK-RAS*, and Hippo pathways, with C1 uniquely enriched in *WNT* and C2 in *PI3K* pathways (Figures 2C and 2D). Despite these pathway differences, there was no significant difference in the overall mutation burden between the two clusters (Figure 2E). However, the specific oncogenic signatures differed, with C2 showing higher mutation rates in *PIK3CA*, *OPA1*, *XIRP1*, and *PTEN*, while C1 was more frequently mutated in *TP53, WDR87, ANK1*, and *WDFY4* (Figures 2F and 2G).

### Tumor microenvironment differences in metabolic clusters

**Figure 3:**
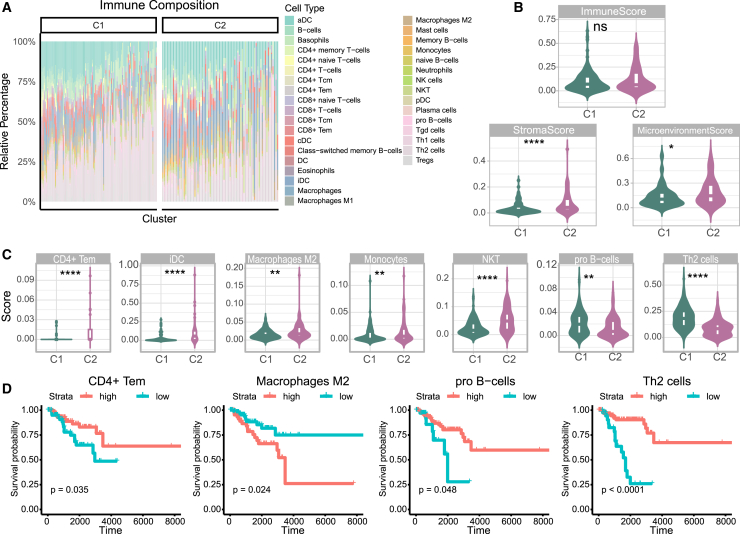
Tumor Microenvironment (TME) Alterations Between TNBC Metabolic Subclusters (A) Bar plot of tumor immune composition in TNBC metabolic subclusters C1 and C2. (B) Violin plot comparing immune score, stromal score, and microenvironment score between TNBC metabolic subclusters. (C) Violin plot of significant immune cell scores between TNBC metabolic subclusters. (D) Kaplan-Meier (KM) plot for significant immune cell scores. ∗*p* < 0.05, ∗∗*p* < 0.01, ∗∗∗*p* < 0.001, ∗∗∗∗*p* < 0.0001.

To explore differences in the tumor microenvironment between C1 and C2, we employed xCell analysis to reconstruct immune cell proportions (Figure 3A). C2 exhibited a higher stromal score compared with C1, although the overall immune score did not differ significantly between the two clusters (Figure 3B). The microenvironment score, which combined immune and stromal scores, reflected the same pattern as the stromal score (Figure 3B). In terms of specific immune cell types, *CD4*+ effector memory T cells (Tem), immature dendritic cells (iDC), M2 macrophages, monocytes, and natural killer T cells (NKT) were more abundant in C2, whereas pro-B cells and type 2 T-helper cells (Th2) were more prevalent in C1 (Figure 3C). Kaplan-Meier (KM) survival analysis revealed that higher levels of *CD4*+ Tem, pro-B cells, and Th2 cells were associated with improved prognosis, whereas elevated levels of M2 macrophages were linked to poorer outcomes (Figure 3D).

### Core metabolic signatures in TNBC metabolic subclusters

**Figure 4:**
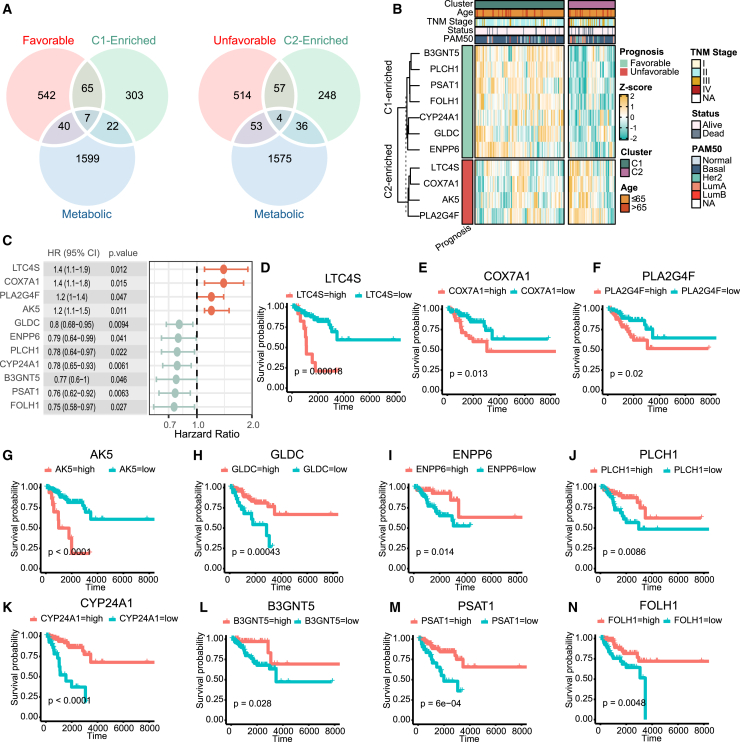
Key metabolic signature of TNBC (A) Venn diagram showing the overlap of metabolic genes and survival-related genes enriched in TNBC subcluster C1 (left) and C2 (right). (B) Heatmap of key metabolic signatures in TNBC mRNA profiles, with prognostic information annotated on the left based on univariate Cox hazard analysis. (C) Forest plot of key TNBC metabolic signatures, where the hazard ratio of PGNG genes was calculated using univariate Cox hazard analysis. (D–N) Kaplan-Meier (KM) plots of key TNBC metabolic signatures, including *LTC4S* (D), *COX7A1* (E), *PLA2G4F* (F), *AK5* (G), *GLDC* (H), *ENPP6* (I), *PLCH1* (J), *CYP24A1* (K), *B3GNT5* (L), *PSAT1* (M), and *FOLH1* (N).

To identify key metabolic signatures in TNBC, we cross-referenced metabolic genes, prognostic markers, and differentially expressed genes between C1 and C2. Given that C1 was associated with better prognosis, we focused on the genes enriched in C1 with protective effects, and the genes enriched in C2 with detrimental effects (Figure 4A). This approach identified seven protective and four harmful metabolic genes (Figure 4A). The heatmap confirmed the differential expression of these genes between the clusters (Figure 4B). The hazard ratios (HRs) for harmful genes ranged from 1.2 to 1.4, while HRs for protective genes ranged from 0.75 to 0.8 (Figure 4C). KM survival plots further validated the prognostic relevance of these genes, with higher expression of *LTC4S*, *COX7A1*, *PLA2G4F*, and *AK5* correlating with poorer survival (Figures 4D–4G), while higher levels of *GLDC*, *ENPP6*, *PLCH1*, *CYP24A1*, *PSAT1*, and *FOLH1* were linked to improved survival (Figures 4H–4N).

### Cellular sources of key metabolic genes

**Figure 5:**
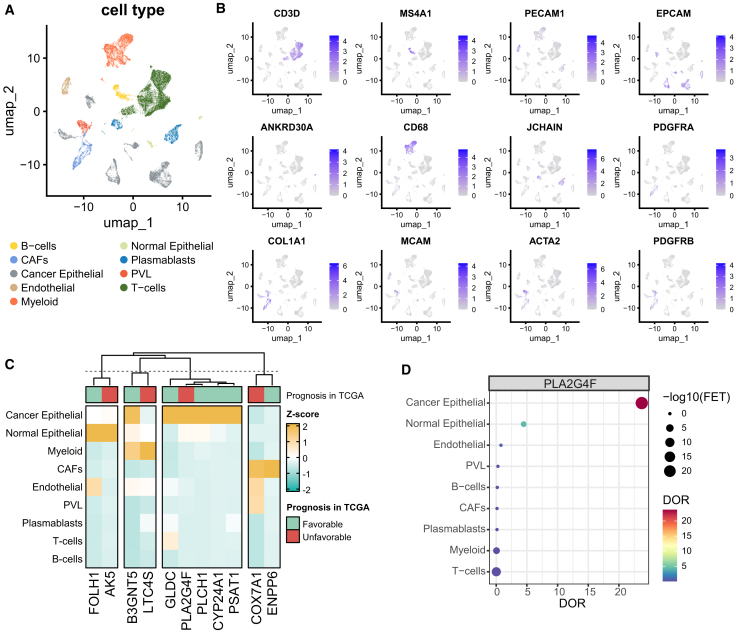
Cellular source at single-cell level of key TNBC metabolic signatures identified in TCGA cohort (A) UMAP plots of TNBC scRNA-seq cells, colored by cell type. (B) UMAP plots showing the expression of cell marker genes for normal epithelial cells (*EPCAM*, *ANKRD30A*), cancer epithelial cells (*EPCAM*), T cells (*CD3D*), myeloid cells (*CD68*), B cells (*MS4A1*), plasmablasts (*JCHAIN*), endothelial cells (*PECAM1*), cancer-associated fibroblasts (*PDGFRA*, *COL1A1*), and perivascular-like cells (*MCAM*, *ACTA2*, *PDGFRB*). Expression levels were log-normalized. (C) Heatmap displaying the scaled diagnostic odds ratio (DOR) scores of key metabolic signatures in the TNBC scRNA-seq dataset, with prognostic information from the TCGA TNBC cohort annotated at the top. (D) Dot plot of *PLA2G4F* DOR scores in the TNBC scRNA-seq dataset, where dot size represents the expression percentage within each cell type.

Given that malignant cells are primarily responsible for reprogramming the tumor microenvironment metabolism, identifying candidate targets that are specifically expressed in these cells is crucial for effective therapeutic intervention. To pinpoint the cellular origins and expression specificity of key metabolic signatures in TNBC, we analyzed an independent breast cancer scRNA-seq dataset. After quality control, normalization, clustering, and annotation, 42,512 cells from 10 TNBC patients were categorized into nine cell types based on canonical lineage markers (Figures 5A and 5B). Using the differential expression ratio (DOR) method, we assessed cell type specificity of the key metabolic genes. Surprisingly, these genes exhibited notable cell specificity. Specifically, *FOLH1* (DOR = 15.3) and *AK5* (DOR = 24.8) were predominantly expressed in normal epithelial cells, while *GLDC* (DOR = 3.3), *PLA2G4F* (DOR = 23.7), *PLCH1* (DOR = 15.3), *CYP24A1* (DOR = 33.1), and *PSAT1* (DOR = 10.9) were mainly expressed in cancer epithelial cells (Figures 5C and 5D). Additionally, myeloid cells were the primary source of *LTC4S* (DOR = 7.34), while cancer-associated fibroblasts (CAFs) were the main source of *COX7A1* (DOR = 14.5) and *ENPP6* (DOR = 43.2) (Figure 5C).

### Suppression of *PLA2G4F* inhibited TNBC cell proliferation and reprogrammed lipid metabolism

**Figure 6:**
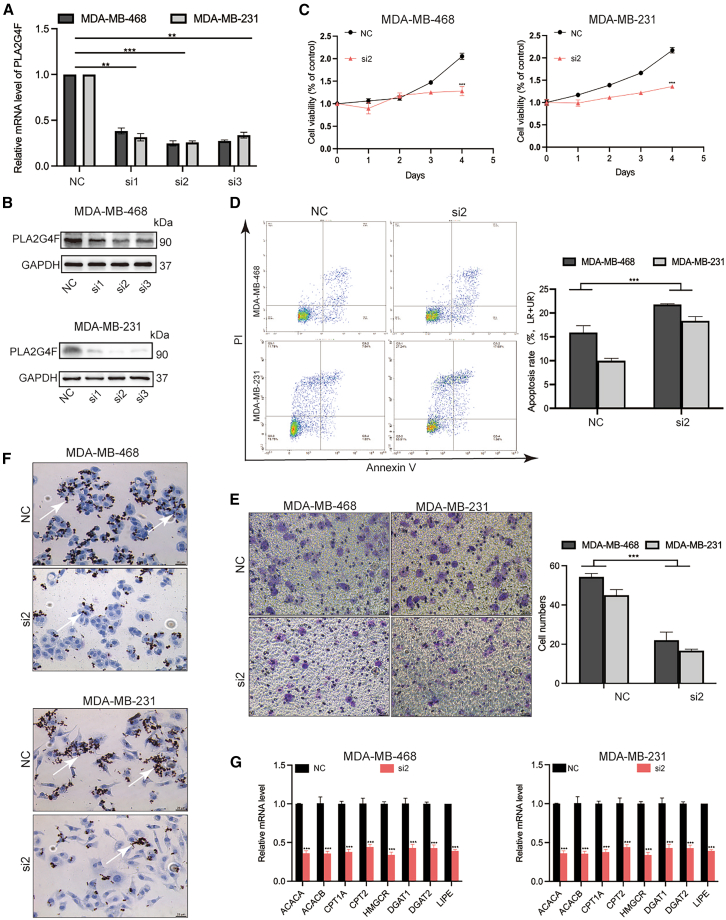
Targeting *PLA2G4F* inhibited the proliferation, induced apoptosis by reprogramming lipid metabolism in TNBC cells (A and B) The relative expression (A) and protein level (B) of *PLA2G4F* was tested after antisense inhibition in TNBC cell lines of MDA-MB-468 and MDA-MB-231. (C) After being transfected with *PLA2G4F* antisense inhibitor or scramble inhibitor, the cell viability of MDA-MB-468 and MDA-MB-231 cells were tested at indicated time points by CCK8 assay. (D) The level of apoptosis of MDA-MB-468 and MDA-MB-231 cells transfected with *PLA2G4F* antisense inhibitor or scramble inhibitor was analyzed by AnnexinV and PI staining by flow cytometry. (E) The transwell assay was used to analyze the migration of transfected MDA-MB-468 and MDA-MB-231. Cell migration number was measured by ImageJ. ∗∗∗*p* < 0.001. (F) The oil red staining for qualitative lipid accumulation of *PLA2G4F* inhibition in MDA-MB-468 (upper panel) and MDA-MB-231 (bottom panel) cells. White arrows indicate positive oil red staining. (G) The relative expression of lipid expression after *PLA2G4F* inhibition in MDA-MB-468 (left panel) and MDA-MB-231 (right panel) cells. ∗∗*p* < 0.01, ∗∗∗*p* < 0.001.

Given the specific expression of *PLA2G4F* in TNBC epithelial cells and its association with poor prognosis, we investigated its oncogenic effects and role in lipid metabolism reprogramming *in vitro*. Three candidate small interfering RNAs (siRNAs) were used to selectively inhibit *PLA2G4F* at both transcriptional and translational levels in the TNBC cell lines MDA-MB-231 and MDA-MB-468 (Figures 6A and 6B). Among these, siRNA2 was the most effective in reducing *PLA2G4F* expression in both cell lines and was selected for further studies (Figures 6A and 6B). Inhibition of *PLA2G4F* significantly reduced cell proliferation and increased apoptosis in both TNBC cell lines (*p* < 0.001, Figures 6C and 6D). Additionally, *PLA2G4F* suppression significantly impaired cancer cell migration (*p* < 0.001, Figure 6E).

Considering the role of *PLA2G4F* in lipid metabolism, we next explored its impact on lipid metabolic reprogramming in TNBC cells. The inhibition of *PLA2G4F* resulted in a marked reduction in lipid formation (Figure 6F). We also assessed the effects of *PLA2G4F* inhibition on key lipid metabolism genes, including those involved in FAS (*ACACA*, *ACACB*), fatty acid oxidation (*CPT1A, CPT2*), cholesterol synthesis (*HMGCR*), triglyceride synthesis (*DGAT1*, *DGAT2*), and lipolysis (*LIPE*). Inhibition of PLA2G4F broadly suppressed the expression of these lipid metabolism genes (Figure 6G), underscoring the critical role of *PLA2G4F* in regulating lipid metabolism in TNBC cells.

### Antisense inhibition of *PLA2G4F* reduces glucose metabolism and the *AKT/PI3K* signaling pathway

**Figure 7:**
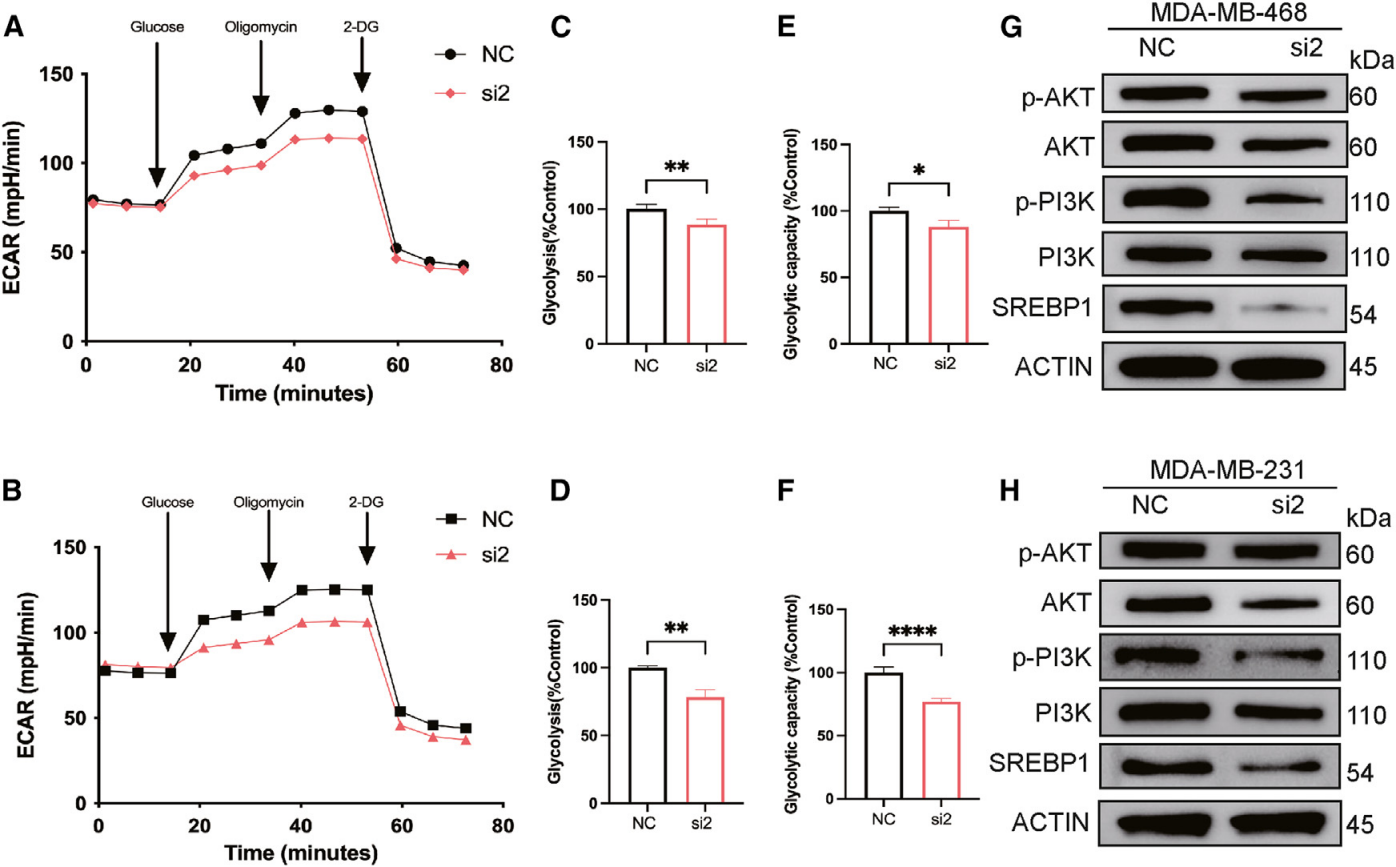
Antisense inhibition of *PLA2G4F* reduces glucose metabolism and suppresses the *AKT/PI3K/SREBP1* signaling pathway in TNBC cells (A and B) Representative ECAR profiles of MDA-MB-468 (A) and MDA-MB-231 (B) cells measured using the Glycolysis Stress Test. Sequential injections of glucose, oligomycin, and 2-deoxyglucose (2-DG) were applied to assess glycolytic function. (C and D) Comparison of basal ECAR levels between si-*PLA2G4F* and control groups in MDA-MB-468 (C) and MDA-MB-231 (D) cells. (E and F) Comparison of glycolytic capacity between si-*PLA2G4F* and control groups in MDA-MB-468 (E) and MDA-MB-231 (F) cells. (G and H) Western blot analysis of *AKT, phospho-PI3K*, and *SREBP1* protein expression in control and si-PLA2G4F-treated cells in MDA-MB-468 (G) and MDA-MB-231 (H). β-actin was used as a loading control. Data are presented as mean ± SEM from three independent experiments. ∗∗*p* < 0.01, ∗∗∗*p* < 0.001.

To investigate the impact of *PLA2G4F* on glycolysis, we performed extracellular acidification rate (ECAR) experiments, a widely used approach to evaluate glycolytic function in living cells. Interestingly, both glycolysis and glycolytic capacity were significantly reduced following antisense inhibition of *PLA2G4F* in MDA-MB-231 and MDA-MB-468 cells (Figures 7A–7F). These findings indicate that *PLA2G4F* plays a critical role in the regulation of glycolysis. In addition, we found that *PLA2G4F* inhibition significantly suppressed the *AKT/PI3K* signaling pathway, as evidenced by a notable decrease in *phospho-PI3K* levels (Figures 7G and 7H). Interestingly, while *phospho-AKT* levels exhibited only a limited reduction, the observed downregulation of *SREBP1* protein further supports the notion that PLA2G4F acts as a key regulator of both glycolysis and the *AKT/PI3K* signaling pathway (Figures 7G and 7H). The consistent results obtained in both MDA-MB-231 and MDA-MB-468 cells provide strong evidence for the pivotal role of *PLA2G4F* in metabolic and signaling processes critical for tumor cell progression.

## Discussions

In this study, we systematically explored the role of metabolic reprogramming in TNBC using a multi-omics approach that integrates bulk RNA sequencing data with scRNA-seq analysis. Our findings revealed significant heterogeneity within the metabolic landscape of TNBC, identifying distinct metabolic subclusters closely associated with patient prognosis, lipid and glucose metabolism, and the tumor microenvironment. By characterizing the cell-specific expression of key metabolic genes, we have laid the foundation for understanding the complex interplay between metabolism and malignancy in TNBC. Our study provides a comprehensive insight into TNBC metabolic reprogramming and identifies potential therapeutic targets for further clinical investigation.

Among the key genes identified, *PLA2G4F* emerged as a particularly promising target due to its specific expression in malignant cells and strong association with oncogenic processes. Previous studies have implicated *PLA2G4F* in various cancers, including prostate cancer and endometrial carcinoma, where it promoted tumor progression through lipid metabolic pathways.^16^^,^^17^ Additionally, *PLA2G4F* has been reported to exhibit calcium-dependent phospholipase and lysophospholipase activities, with potential roles in membrane lipid remodeling and the biosynthesis of lipid mediators.^18^ Our results not only confirmed the involvement of *PLA2G4F* in TNBC but also demonstrated its broader impact on lipid metabolism, including the regulation of FAS, oxidation, cholesterol synthesis, and triglyceride synthesis. The inhibition of *PLA2G4F* significantly reduced lipid accumulation in TNBC cell lines, underscoring its role as a critical regulator of metabolic reprogramming in cancer.

Lipid metabolism plays a crucial role in TNBC progression.^19^ Increased FAS contributes to the generation of structural lipids necessary for cell membrane formation, leading to lipid accumulation during cell proliferation.^20^^,^^21^^,^^22^ Moreover, alterations in lipid metabolism in cancer cells have implications for immune evasion. Studies on breast and prostate cancer cell lines have shown that activation of PI3K kinase promotes immunoresistance, particularly through the expression of programmed cell death ligand 1 (*PD-L1*), a negative regulator of T cell function.^23^ Our study further illuminates the relationship among *PLA2G4F*, lipid metabolic reprogramming, and the immune microenvironment in TNBC. Differential expression of *PLA2G4F* was associated with changes in immune cell composition, particularly affecting cell types such as CD4+ Tem, iDCs, and M2 macrophages. These findings suggested that *PLA2G4F* might influence the immune landscape of TNBC, potentially contributing to an immunosuppressive microenvironment that supports tumor growth. This connection between *PLA2G4F* and the immune microenvironment highlights its potential as a dual target for both metabolic and immunotherapy strategies.

While the tumor mutation burden (TMB) between C1 and C2 is similar, the distinct differences in the frequency of specific mutation types and genes have notable implications for the immune microenvironment. In C2, higher mutation rates in *PIK3CA* and *PTEN* likely contribute to a more immune-suppressive microenvironment. Tumor cells with *PTEN* loss are associated with increased recruitment of myeloid-derived suppressor cells (MDSCs), regulatory T cells (Tregs), and M2 macrophages, along with impaired interferon signaling pathways.^24^ Additionally, overactivation of the *PI3K* pathway or *PTEN* loss can induce PD-L1 overexpression, which suppresses T cell proliferation.^25^ In contrast, *TP53*, which shows higher mutation frequency in C1, plays a dual role in immune regulation. On the one hand, *TP53* suppresses pro-inflammatory factors, such as *NF-κB* and *STAT3*, to maintain tissue homeostasis and limit excessive immune responses. On the other hand, *TP53* enhances antitumor immunity by upregulating MHC class I expression and promoting the recognition of tumor antigens.^26^^,^^27^ Additionally, *TP53* mutations have been linked to increased pro-B cell proportions, as observed in our analysis, supporting its unique immunomodulatory role. Our results demonstrated that *PLA2G4F* is predominantly expressed in malignant cells and serves as a key regulator of glucose and lipid metabolism. Based on these findings, we hypothesize that *PLA2G4F* may influence the tumor microenvironment through metabolic mediators, such as lactate and ω3 polyunsaturated fatty acid (PUFA)-derived anti-inflammatory lipid mediators.^28^^,^^29^

Finally, the expression specificity of *PLA2G4F* in malignant cells, combined with its pivotal role in metabolic reprogramming and immune modulation, makes it an attractive therapeutic target. Targeting *PLA2G4F* could disrupt the metabolic underpinnings of TNBC while simultaneously modulating the immune microenvironment to enhance antitumor responses. Given the current lack of effective targeted therapies for TNBC, *PLA2G4F* represents a novel and promising candidate for drug development. Future studies should focus on preclinical validation of *PLA2G4F* inhibitors and explore their efficacy in combination with existing treatments, such as chemotherapy and immunotherapy.

However, there are several limitations to this study. First, our experiments were conducted *in vitro*, and further *in vivo* testing is required. Interference with *PLA2G4F* expression may influence tumor growth by remodeling the tumor microenvironment, which should be assessed using histological approaches. Second, while we observed changes in gene expression and limited metabolite levels, more comprehensive research is needed to confirm these changes at the metabolite level both *in vivo* and *in vitro*, including key molecules such as fatty acids. A metabolomic analysis would improve our understanding of the regulatory mechanisms underlying the metabolic switch mediated by *PLA2G4F*. Finally, the potential upstream regulation of *PLA2G4F*, including epigenetic modifications linked to metabolic reprogramming in TNBC, should be the focus of future research.

## Materials and methods

### TNBC data acquisition and processing

RNA sequencing data and somatic mutation profiles for TCGA BRCA were acquired using the R package TCGABiolinks.^30^ The gene ID conversion and gene biotype annotation were performed using the R package biomaRt.^31^ The TNBC samples were defined by previous research according to the bimodal expression of ESR1, PGR, and ERRBB2.^32^ Clinical data, including survival information and tumor stage, were obtained from the Genomic Data Commons (GDC) Data Portal (https://gdc.cancer.gov/about-data/publications/pancanatlas).

### Bioinformatics analysis

Consensus clustering was conducted using the R package ConsensusClusterPlus.^33^ The number of clusters (k) was evaluated from 2 to 9, with the final clustering determined by examining the CDF curves of the consensus score matrix and the PAC statistics. Survival-related and metabolic genes were incorporated into the consensus clustering process.

Differentially expressed genes (DEGs) were identified using the R package limma, with thresholds set at |log2FoldChange| > 1 and *p* value <0.05.^34^ Gene enrichment analysis was carried out using the “enrichGO” and “enrichKEGG” functions in the R package clusterProfiler, and visualized using the R package enrichplot.^35^

Survival analysis, including Cox proportional hazards and Kaplan-Meier (KM) analyses, was performed using the R package survival. For KM analysis, the cutoff value was determined using the surv_cutpoint function from the R package survminer, separating samples into high and low expression groups. KM survival curves were visualized using the “ggsurvplot” function from survminer.

Somatic mutation analysis of TNBC tumors was performed and visualized with the R package maftools.^36^ Mutations, including missense, silent, nonsense, frameshift/in-frame insertions and deletions, and uninterrupted mutations, were counted, while synonymous mutations were excluded. Tumor mutation burden (TMB) was calculated based on the total number of somatic mutations. Pathway enrichment analysis, focusing on known oncogenic or custom pathways, was performed using the “pathways” function. Mutational profile differences between metabolic TNBC subclusters were analyzed using the “mafCompare” function. The tumor microenvironment estimation was applied by R package xCell.^37^

scRNA-seq data for BRCA was sourced from a published study and accessed via the GEO database (accession: GSE176078). Cells from 10 TNBC patients were selected for subsequent analysis. The scRNA-seq data were processed using the R package Seurat, involving quality control, normalization, scaling, dimensional reduction, and clustering. Cell types were identified using canonical gene markers, including T cells (*CD3D*), B cells (*MS4A1*), endothelial cells (*PECAM1*), normal epithelial cells (EPCAM, ANKRD30A), cancer epithelial cells (*EPCAM*), myeloid cells (*CD68*), plasmablasts (*JCHAIN*), cancer-associated fibroblasts (CAFs) (*PDGFRA*, *COL1A1*), and perivascular-like cells (*PVL*) (*MCAM*, *ACTA2*, *PDGFRB*). Normal and cancer epithelial cell annotations were obtained from the original research, and cell type distinction was performed using the R package inferCNV.^38^

The spatial distribution of specific genes was assessed using the diagnostic odds ratio (DOR).^39^ For each gene, DOR was calculated by binarizing expression values, where a normalized expression value >0 was considered positive, and zero expression was considered negative. To prevent undefined values, a pseudocount of 0.5 was added, with the DOR calculated as follows:

(Equation 1) $$DOR=\frac{\left(\text{TP}+0.5\;\right)\;/\left(\text{FP}+0.5\right)}{\left(FN+0.5\;\right)\;/\left(TN+0.5\right)}$$

Here, True Positives (TP) represent the number of cells within the group expressing the gene, False Positives (FP) represent the number of cells outside the group expressing the gene, False Negatives (FN) represent the number of cells within the group not expressing the gene, and True Negatives (TN) represent the number of cells outside the group not expressing the gene.

### Cell culture and lentivirus transfection

The MDA-MB-231 and MDA-MB-468 TNBC cell lines were obtained from the National Collection of Authenticated Cell Cultures of China. These cells were cultured in Dulbecco’s modified Eagle’s medium (DMEM) supplemented with 10% fetal calf serum (FCS), 1% sodium pyruvate, and 1% penicillin/streptomycin (Thermo, USA). All cultures were maintained at 37°C in a humidified incubator with 5% CO_2_. Small interfering RNAs (siRNAs) targeting PLA2G4F, as well as a negative control, were provided by GenePharma (China). The sequences (sense, 5′–3′) were as follows:

si-1 GGCUACAGUACUACACUCATT, si-2 CCCUAUGGCAUGAUGAACUTT, si-3 GGGAAACCUACCCAUACUATT. The inhibitory effect of siRNA on PLA2G4F expression was confirmed at the mRNA level by qPCR and at the protein level by western blot analysis.

### qPCR

Total RNA of the tissue and cells was extracted using RNA Isolator Total RNA Extraction Reagent (Vazyme, China), and the RNA was reverse transcribed by PrimeScript RT Master Mix (TaKaRa, Japan), according to the manufacturer’s instructions. The qPCR assay was tested by SYBR Green Mix (Life Technology, USA) and quantified by ABI 7500 real-time PCR system (Applied Biosystems, USA). The relative expression of each gene was normalized to Actin by the 2-ΔCt method. The specific primer sequences are listed in Table 1.

**Table 1:** Primer sequences (5′–3′)

Gene name	Forward	Reverse
*PLA2G2F*	GATCTGTTACCTGCAAGGTATGTGG	TTCTGGCAGTCGTCTGTGATATTC
*GAPDH*	ACAGCCTCAAGATCATCAGCAATGC	GATGGCATGGACTGTGGTCATGAGT
*ACACA*	TCACACCTGAAGACCTTAAAGCC	AGCCCACACTGCTTGTACTG
*ACACB*	AGAAGACAAGAAGCAGGCAAAC	GTAGACTCACGAGATGAGCCA
*CPT1A*	ATCAATCGGACTCTGGAAACGG	TCAGGGAGTAGCGCATGGT
*CPT2*	CATACAAGCTACATTTCGGGACC	AGCCCGGAGTGTCTTCAGAA
*HMGCR*	CGTGGAATGGCAATTTTAGGTCC	ATTTCAAGCTGACGTACCCCT
*DGAT1*	GGTCCCCAATCACCTCATCTG	TGCACAGGGATGTTCCAGTTC
*DGAT2*	ATTGCTGGCTCATCGCTGT	GGGAAAGTAGTCTCGAAAGTAGC
*LIPE*	GACCCCTGCACAACATGATG	TGAGCAGCACCCTTTGGATG

### Western blot

Western blot was carried out as described before.^40^ The primary antibody was used as follows: *PLA2G4F* (#PA5-31735, Thermo Fisher), *GAPDH* (#2118, CST), *PI3K* (**#4249,** CST), *p-PI3K* (**#13857**, CST), *AKT* (**#9272,** CST), *p-AKT* (**#9271,** CST), *SREBP1* (Thermo Fisher Scientific, USA), and *β-actin* (#4967, CST).

### Transwell migration assay

The transfected cells were seeded in the upper chamber of 24-well plates in 200 μL medium without FBS and the bottom chamber was added by 500 μL complete medium with 10% FBS. Then the cells in each well were fixed after migration for 48 h, followed by staining with hematoxylin. The cells inside the chamber were removed, while those that had migrated to the outer surface of the membrane were imaged by microscope. The experiments were performed three times independently.

### Cell viability assays

Cells subjected to different transfections were seeded at 5,000 cells/well in 96-well plates in triplicate. Plates were incubated at 37°C, and cell viability was measured every 12 h using the Cell Counting Kit-8 (CCK8, Abbkine, China). Optical absorbance at 450 nm was determined using a microplate reader (Bio-Rad Laboratories, USA). Relative cell viability was normalized to control wells.

### Cell apoptosis assay

Apoptosis levels in cells were measured using flow cytometry. Tumor cells were transfected with siRNA and incubated for 48 h. The cells were then collected, stained using the Annexin V/FITC kit (Beyotime, China), and analyzed by flow cytometry (BD, USA).

### Oil red O staining

Cell lines were seeded in 96-well plates as described above. After transfection with siRNA for 24 h, the oil red O staining kits (Sigma) were used to stain fat droplets in the wells. In brief, cells were washed with PBS three times and fixed for 30 min. Discarding the liquid, 60% isopropanol was supplied to the cells for 30 s. Then oil red O staining solution was added into each well for 30 min and hematoxylin was incubated subsequently. Finally, the plates were washed in running water for 1 min and the images were taken by microscope.

### Extracellular acidification rate test

Extracellular acidification rate (ECAR) was measured in real time with Glycolysis Stress Test Kit and Mito Stress Test Kit, respectively, using the Seahorse XFe96 Analyzer (Agilent Technologies) following the manufacturer’s instructions. Data were normalized by cell numbers that were measured by the YO-PRO-1 Assay (Thermo Fisher Scientific).

### Statistical analysis

R (Version 4.1.2) was used for all statistical tests. Spearman correlation analyses were conducted using R. The significance of qPCR results was determined using a Student’s t test, assuming a normal distribution. *p* values of multiple tests were adjusted by false discovery rate (FDR). All *p* values and adjusted *p* values were considered significant if less than 0.05.

## Data availability

Public data of the BRCA RNA sequencing profile and clinical data are available on the TCGA Research Network portal (https://portal.gdc.cancer.gov/). The scRNA-seq data for BRCA were sourced from a published study and accessed via the GEO database (accession: GSE176078).

## Acknowledgments

We extend our appreciation to all the researchers who generously shared the data utilized in this study. No animal studies are presented in this manuscript. The studies involve no human participants. This work was supported by the 10.13039/501100001809National Natural Science Foundation of China (Grant Nos. 82003077 and 82472363) and program of Jiangsu Commission of Health Department (Grant No.H2019070).

## Author contributions

X.T.: Conceptualization, Data curation, Formal analysis, Methodology, Investigation, Visualization, Writing – original draft. N.J.: Methodology, Validation, Writing – original draft. Y.K.: Conceptualization, Methodology, Validation, Writing – original draft. S.C. and F.Y.: Conceptualization, Methodology, Supervision, Writing – review and editing.

## Declaration of interests

The authors declare no competing interests.
